# Early weight gain after diagnosis may have an impact on remission status in children with new‐onset type 1 diabetes mellitus

**DOI:** 10.1111/1753-0407.13455

**Published:** 2023-08-12

**Authors:** Dicle Canoruc Emet, Hande Nur Karavar, Onur Gozmen, Arife Aslan Agyar, Yağmur Ünsal, Merve Canturk, Pınar Cengiz, Dogus Vuralli, Z. Alev Ozon, E. Nazlı Gonc

**Affiliations:** ^1^ Department of Pediatrics, Division of Endocrinology Hacettepe University Ankara Turkey

**Keywords:** BMI, remission, type 1 diabetes, weight change, BMI, 缓解, 1型糖尿病, 体重变化

## Abstract

**Background:**

Residual beta‐cell function and improvement in insulin sensitivity by reversal of glucose toxicity are two phenomena thought to be related to partial remission (PR). Body fat mass is the major determinant of insulin sensitivity. The aim of this study is to investigate the relationship between the rate of body weight gain after diagnosis of type 1 diabetes mellitus (T1DM) and other clinical factors for the development and duration of PR.

**Methods:**

Children (2–16 years) with new‐onset T1DM (*n* = 99) were grouped into remitters and non‐remitters by using insulin dose‐adjusted glycosylated hemoglobin (HbA1c) values. Laboratory and clinical data as well as daily insulin requirement per kilogram of body weight at diagnosis and each visit were recorded, and the duration of PR was determined. Changes in body mass index standard deviation score (BMI‐SDS) were calculated by the auxological data collected every 6 months.

**Results:**

There were 47 remitters (47.5%) and 52 (52.5%) non‐remitters. The mean increase in BMI‐SDS at the first 6 months of diagnosis was higher in the non‐remitters than in the remitters (*p* = 0.04). Duration of PR was negatively correlated with the change in BMI‐SDS between 6 and 12 months after diagnosis. Male sex, younger age, prepubertal status, and lower HbA1c were predictors of remission, among which male sex had the highest chance by multivariate regression.

**Conclusions:**

Early rapid weight gain after diagnosis of T1DM may play a role in the lack of remission and shorter duration of PR. Interventions to prevent early rapid weight gain can maintain the development and prolongation of remission.

## Graphical abstract

**Highlights**

- Weight gain with insulin therapy in the early period may be negatively related to the occurrence and duration of remission by altering insulin sensitivity.
- Interventions to prevent rapid weight gain and conserve insulin sensitivity in the early stages of type 1 diabetes mellitus may be beneficial for the development and maintenance of remission.
- This is the first study, as far as we have investigated the English literature, that shows the significant association between early weight gain after diagnosis and the emission phase. Our study will provide an idea and guide to prospective studies.

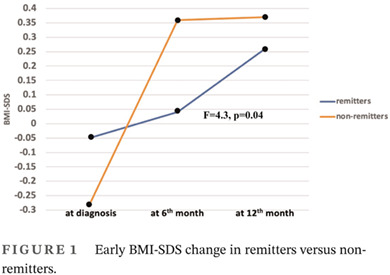

## 摘要

背景:残余β细胞功能和通过逆转葡萄糖毒性改善胰岛素敏感性被认为是与部分缓解(PR)相关的两种现象。体脂质量是胰岛素敏感性的主要决定因素。本研究旨在调查1型糖尿病(T1DM)诊断后体重增加率与其他临床因素之间的关系，以确定PR的发生和持续时间

方法:将新发T1DM儿童(2‐16岁)(n=99)根据胰岛素剂量调整后的HbA1c值分为缓解组和非缓解组。记录实验室和临床数据以及诊断时和每次随访时每千克体重的每日胰岛素需求量，确定PR持续时间。以每六个月收集的生长发育数据，计算体重指数标准差评分(BMI‐SDS)的变化

结果:47名患者(47.5%)病情缓解，52名患者(52.5%)病情未缓解。诊断后前6个月，病情未缓解患者的BMI‐SDS平均增加值高于病情缓解患者(p=0.04)。PR持续时间与诊断后第6个月至第12个月BMI‐SDS的变化呈负相关。多元回归分析显示男性、年轻、青春期前状态和较低的HbA1c是病情缓解的预测因素，其中男性的可能性最大

结论:T1DM诊断后早期体重快速增加可能在对难以缓解和PR持续时间较短起作用。预防早期体重快速增加的干预措施可能有助于维持和延长缓解

## 1 INTRODUCTION

Type 1 diabetes mellitus (T1DM) is a result of immune‐mediated beta‐cell destruction leading to absolute insulin deficiency.^1^ Although the damage is progressive and irreversible, the diagnosis of T1DM may be followed by a special period called “partial clinical remission (PR),” which is characterized by decreased insulin requirement and good glycemic control. Many different definitions have been proposed to designate PR.^2^ In order to standardize the definition, insulin dose‐adjusted hemoglobin A1c (IDAA1c) was suggested initially in 2009 and is generally accepted as gold standard today.^3^

The peak prevalence of PR usually occurs in the first 3–6 months. Afterward, the PR rate declines, ranging from 0%–20% at 6 months and only up to 10% at 12 months.^2^, ^4^ PR is associated with improved glycemic control and reduced risk for acute and chronic complications.^5^ In addition, PR is a real‐life model giving a chance to examine factors and new therapeutic options for conserving and prolonging residual beta‐cell function and good glycemic control. Predictors and physiology underpinning remission and its duration have not been fully elucidated yet. However, recovery of residual beta‐cell function and improvement in insulin sensitivity by reversal of glucose toxicity are the two main phenomena suggested to be associated with remission.^2^, ^3^

Insulin resistance is the main pathophysiologic defect that leads to type 2 diabetes mellitus. In recent years, however, it has been understood that insulin resistance is also a feature of T1DM.^6^ In patients with T1DM, insulin requirement is individualized according to both residual insulin secretion and insulin sensitivity. Almost all studies examining the role of insulin sensitivity in the course of T1DM in the literature are related to the period after remission.^7^, ^8^ Recently, Niedzwiecki et al. showed that non‐remitters had higher insulin resistance in comparison to remitters in the long term.^5^ In another study, a negative correlation of C‐terminal cross‐linked telopeptide (CTX), which is considered to be associated with insulin sensitivity, and the increase of IDAA1c in the first year of T1DM was found to be independent of C‐peptide decline. Therefore, these findings suggested that remission may be related to insulin sensitivity rather than C‐peptide production.^9^

The main and most powerful determinant of insulin resistance is body fat content, which can be measured indirectly by body mass index (BMI).^10^ A significant increase in BMI is frequently observed in patients with newly diagnosed T1DM in the first few months of insulin therapy, concurring with the emergence of remission.^11^ Thus, early and significant weight gain following intensive insulin therapy may lead to insulin resistance and may have an impact on the occurrence of remission.

Therefore, our first aim is to examine the relationship between the early change in BMI standard deviation score (SDS) and the development and duration of remission in children and adolescents with newly diagnosed T1DM. Secondly, we aim to investigate other possible predictors for the development and duration of remission.

## 2 METHODS

This is a retrospective study conducted at the pediatric endocrinology clinic. The study protocol was approved by the local clinical research ethics committee (GO22/382).

### 2.1 Participants and treatment protocol

The participants of the study consisted of 2–16‐year‐old children with T1DM, who had been diagnosed between January 1, 2016, and December 30, 2020. Follow‐up records for 1 year after diagnosis were reviewed retrospectively.

The diagnosis of T1DM was based on International Society for Pediatric and Adolescent Diabetes (ISPAD) 2018 guidelines. Classical symptoms of diabetes at onset and presence of at least one of the diabetes‐associated autoantibodies, low C‐peptide with simultaneous high blood glucose, presentation with diabetic ketoacidosis (DKA) or ketosis, age at diagnosis >12 months, and lack of an autosomal dominant family history of diabetes were considered as possible indicators for T1DM. Only anti‐GAD and anti‐islet cell antibody measurements are available in our laboratory. All patients enrolled in the study had at least one positive antibody. Both anti‐GAD and anti‐islet cell autoantibodies were positive in 39 patients, and in 60 patients only one autoantibody was positive. Patients who had been exposed to drugs with β‐cell toxicity or causing insulin resistance and those with diseases that can affect body weight gain, such as celiac disease, were not included.

Patients are hospitalized at diagnosis for 7–10 days, and treatment of each patient with T1DM is based on established routines at our clinic within the context of the ISPAD guidelines. Treatment of patients presenting with ketoacidosis or ketosis is started with appropriate fluid and intravenous insulin infusion in the intensive care unit. After the resolution of acidosis and ketosis, treatment is shifted to multiple injection therapy (MIT) with insulin lispro (Humolog) at every meal and insulin glargine (Lantus) once daily at dinner time. Both parents of all patients are encouraged to learn the basic rules of diabetes management and self‐monitoring of blood glucose at least eight times a day (before and after each meal and at 12.00 PM and 03.00 AM) by the diabetes team (the endocrinologists, nurses, dieticians, and a clinical psychologist). Following discharge, daily visits are carried out via telemedicine for the first 10 days. Thereafter, control follow‐up visits are planned on the 15th day, monthly for the first 3 months, and then every 3 months for blood sugar control and insulin dose adjustment. Auxological measurements and physical examinations are performed every 6 months, and glycosylated hemoglobin (HbA1c) values are measured every three months. None of the patients in the study was using continuous glucose monitoring or continuous subcutaneous insulin infusion.

### 2.2 Study design

Clinical and laboratory data including age, auxological measurements, pubertal development (prepuberty or puberty), presentation (DKA, ketosis, or hyperglycemia), serum pH, bicarbonate, initial blood glucose, HbA1c, insulin, and C‐peptide at the time of diagnosis were recorded. At the 6th‐ and 12th‐ month follow‐up visits, details of insulin treatment, self‐monitoring blood glucose measurements, HbA1c values, and auxological measurements were noted. The IDAA1c formula, which is HbA1c (%) + [4 × total daily insulin dose (TDD, IU/kg/24 h)], was calculated for each participant at each visit.^3^ Patients were divided into two groups, remitters and non‐remitters, according to IDAA1c value. An IDAA1c value ≤9 at any visit within the first year was regarded as PR,^3^ and the time to completion of PR was also noted to assess the duration of PR. Early BMI‐SDS change was evaluated using the auxological parameters at the time of diagnosis and in the outpatient control at the 6th month after diagnosis. Since the degree of weight loss before diagnosis might influence early weight change, premorbid BMI‐SDS was calculated by the declaration of families of how much weight their children lost before diagnosis, and the change was compared between the two groups. However, since these data were based on verbal statements, it was not included in the statistics for early weight change. The relation between clinical features, laboratory parameters at the time of diagnosis, and early BMI‐SDS change and the development of remission was analyzed. The relationship between 6 and 12 months of weight change and the duration of remission was also examined.

DKA was defined according to ISPAD.^12^ Patients were divided into two groups: DKA and non‐DKA. TDD which is expressed in units (U)/kg/day was calculated as doses of long‐acting plus short‐acting insulins administered daily and divided by the subject's weight in kilograms. HbA1c was measured by high‐performance liquid chromatography using the 723 TOSOH G8 automated glycohemoglobin analyzer (Tosho Bioscience; intrabatch and interbatch coefficient of variation [CV] < 2%, reference range 3%–19%) based on Diabetes Control and Complications Trial standards.

### 2.3 Auxological measurements

Measurements of patients were recorded at each visit. The measurements were taken by the same nurse using the same calibrated wall‐mounted Harpenden stadiometer and a digital scale in the morning, following 8 hours of fasting. BMI was calculated by dividing weight (kilograms) by the square of height (meters). BMI‐SDS were determined according to the LMS (power transformation for normality, median, generalized coefficient of variation) method using 2000 CDC Growth Chart curves.^13^ ΔBMI‐SDS^1^ was defined as the difference of BMI‐SDS at 6 months from that at diagnosis. ΔBMI‐SDS^2^ was defined as the difference of BMI‐SDS at 12 months from that at 6 months.

### 2.4 Statistical analysis

SPSS Statistics version 21.0 (IBM Corp., Armonk, New York) was used for statistical analyses. Pearson's χ^2^ test was used to evaluate categorical variables. The variables were investigated using visual and analytic methods to determine whether they are normally distributed or not. Analysis for normally distributed variables was presented using means and standard deviations, and Student's *t*‐test was used to compare these parameters. Non‐normally distributed variables were compared by using the Mann–Whitney *U* test and presented using medians and minimum (min)‐maximum (max) values as well as means and standard deviations. Predictors for remission and duration of remission were analyzed using univariate and multivariate logistic regression analysis. Repeated measures analysis was used to analyze BMI‐SDS change over time. The effect of remission status, pubertal status, presence of DKA, and sex on the change in BMI‐SDS by time was also investigated by repeated measures analysis of variance. The adjustments according to age, sex, and puberty were done. Since the duration of remission did not comply with normal distribution, the relationship between clinical and laboratory parameters and the duration of remission was analyzed by Spearman's test. An overall 5% type 1 error level was used to infer statistical significance.

## 3 RESULTS

Ninety‐nine children (55 boys) with a mean age of 8.7 ± 3.7 years (boys 8.5 ± 3.7, girls 8.9 ± 3.6 years, *p* = 0.60) were included in the study. Forty‐seven (47.5%) were remitters and 52 (52.5%) were non‐remitters. The clinical and biochemical characteristics of the two groups at diagnosis and their visits are shown in Table 1.

**TABLE 1:** Comparison of clinical and biochemical characteristics between remitters and non‐remitters at diagnosis, 6 months, and 12 months.

		All participants (*n* = 99)	Remitters (*n* = 47)	Non‐remitters (*n* = 52)	*p*
Age (years)		8.7 ± 3.6	7.9 ± 3.4	9.4 ± 3.8	**0.05**
Sex (M/F)		55 (56%)/44 (44%)	34 (72%)/13 (28%)	21 (40%)/31 (60%)	**<0.01**
Puberty					
Prepubertal		73 (74%)	39 (83%)	34 (65%)	**0.04**
Pubertal		26 (26%)	8 (17%)	18 (35%)	
Presentation					
DKA		57 (58%)	26 (55%)	31 (60%)	0.41
Other		42 (42%)	21 (45%)	21 (40%)	
Both antibodies positive Only one antibody positive		39 (39%) 60 (61%)	18 (38%) 29 (62%)	21 (40%) 31 (60%)	0.48
Premorbid weight SDS Premorbid BMI SDS		0.48 ± 1.0 0.58 ± 1.3	0.45 ± 0.9 0.68 ± 1.1	0.52 ± 1.1 0.49 ± 1.4	0.7 0.5
At diagnosis	Auxological measurements				
	Height SDS	0.21 ± 1.08	0.09 ± 1.10	0.25 ± 1.05	0.59
	Weight SDS	0.04 ± 1.22	0.11 ± 1.32	0.02 ± 1.01	0.56
	BMI	16.9 ± 3.1	17.0 ± 3.2	16.8 ± 3.1	0.77
	BMI SDS	−0.25 ± 1.5	−0.05 ± 1.49	−0.28 ± 1.50	0.39
	Laboratory				
	pH	7.21 ± 0.17 7.25 (6.75–7.41)	7.20 ± 0.19 7.25 (6.75–7.41)	7.21 ± 0.16 7.22 (6.84–7.41)	0.69^a^
	Blood glucose (mmol/L)	24.9 ± 7.9 24.3 (9.4–50)	25.4 ± 7.8 23.8 (13.7–48.8)	24.7 ± 7.9 24.3 (9.4–50)	0.51^a^
	HCO_3_ (mmol/L)	14.7 ± 6.2 13.9 (3–25)	14.6 ± 6.2 14.4 (3.9–23.2)	14.7 ± 6.2 12.4 (3–25)	0.91^a^
	Insulin (pmol/L)	15.9 ± 11.8 11.8 (2.2–59)	22.2 ± 40.9 12.7 (2.2–59)	15.9 ± 15.2 11.1 (2.22–51.3)	0.71^a^
	C‐peptide (nmol/L)	0.15 ± 0.13 0.10 (0.03–0.96)	0.15 ± 0.16 0.09 (0.03–0.96)	0.14 ± 0.09 0.10 (0.04–0.38)	0.20^a^
	HbA1c (%)	12.3 ± 2.1	11.8 ± 2.2	12.6 ± 1.9	**0.04**
	TDD (U/kg/day) at discharge	1.13 ± 0.37	1.15 ± 0.42	1.12 ± 0.32	0.64
6th month	Auxological measurements				
	Height SDS	0.35 ± 0.97	0.21 ± 1.0	0.47 ± 0.89	0.92
	Weight SDS	0.38 ± 1.01	0.19 ± 1.2	0.53 ± 0.76	0.51
	BMI	18.2 ± 3.0	17.8 ± 3.4	18.5 ± 2.7	0.32
	BMI‐SDS	0.22 ± 1.03	0.04 ± 1.1	0.36 ± 0.98	0.35
	TDD (U/kg/day)	0.62 ± 0.31	0.48 ± 0.26	0.79 ± 0.29	**<0.01**
	HbA1c (%)	7.2 ± 1.5	6.4 ± 0.79	8.0 ± 1.7	**<0.01**
	ΔBMI^1^ ΔBMI‐SDS^1^	1.4 ± 2.2 0.64 ± 1.05	0.89 ± 2.1 0.41 ± 1.02	1.8 ± 2.2 0.83 ± 1.04	**0.03** **0.04**
12th month	Auxological measurements				
	Height SDS	0.41 ± 0.95	0.23 ± 1.07	0.56 ± 0.83	0.13
	Weight SDS	0.44 ± 0.88	0.27 ± 1.02	0.57 ± 0.74	0.16
	BMI	18.0 ± 2.8	17.4 ± 2.2	18.5 ± 3.1	0.10
	BMI‐SDS	0.32 ± 0.91	0.26 ± 0.99	0.37 ± 0.84	0.67
	TDD (U/kg/24 h)	0.72 ± 0.34	0.55 ± 0.28	0.92 ± 0.31	**<0.01**
	HbA1c (%)	7.7 ± 1.4	7.1 ± 1.0	8.3 ± 1.5	**<0.01**
	ΔBMI^2^	0.42 ± 1.07	0.49 ± 1.03	0.35 ± 1.1	0.55
	ΔBMI‐SDS^2^	0.04 ± 0.59	0.09 ± 0.74	0 ± 0.4	0.42

*Note*: Non‐normally distributed variables are presented as median (min‐max) in addition to mean ± standard deviation. Bold indicates significant value (*p* ≤ 0.05).

Abbreviations: BMI, body mass index; DKA, diabetic ketoacidosis; HbA1c, glycosylated hemoglobin; SDS, standard deviation score; TDD, total daily insulin dose.

^a^ Mann–Whitney *U* test was used to compare variables between remitters and non‐remitters.

The PR developed after a mean period of 3.7 ± 1.3 months following diagnosis. Younger age and lower HbA1c, male sex, prepuberty at diagnosis were the predictors of remission in univariate analysis (Table 2). In the multivariate regression model, the sole predictor of remission was the male sex (Table 2).

**TABLE 2:** Univariate and multivariate logistic model for determinants of remission at diagnosis.

	Odds ratio	95% CI	*p*	Adjusted odds ratio^a^	95% CI	*p*
Age (years)	0.89	[0.80–1.0]	**0.05**	0.9	[0.80–1.0]	0.71
Sex (M)	3.8	[1.65–8.99]	**<0.01**	**3.8**	[1.5–9.6]	**<0.01**
Pubertal status (prepubertal)	2.5	[1.0–6.6]	**0.05**	0.88	[0.70–1.1]	0.30
Clinical presentation (non‐DKA)	1.19	[0.53–2.64]	0.66	‐	‐	‐
Laboratory at diagnosis						
pH	0.88	[0.09–8.70]	0.91	‐	‐	‐
Blood glucose (mg/dL)	1.0	[0.99–1.00]	0.62	‐	‐	‐
HCO_3_ (mmol/L)	0.99	[0.93–1.06]	0.94	‐	‐	‐
Insulin	1.02	[0.93–1.2]	0.37	‐	‐	‐
C‐peptide	1.09	[0.39–3.0]	0.86	‐	‐	‐
HbA1c	0.81	[0.66–1.0]	**0.05**			
Auxology at diagnosis						
Height SDS	0.87	[0.60–1.26]	0.46	‐	‐	‐
Weight SDS	1.07	[0.76–1.5]	0.69	‐	‐	‐
BMI‐SDS	1.1	[0.84–1.44]	0.45	‐	‐	‐

*Note*: Bold indicates significant value (*p* ≤ 0.05).

Abbreviations: BMI, body mass index; DKA, diabetic ketoacidosis; HbA1c, glycosylated hemoglobin; SDS, standard deviation score.

^a^ Adjustments were done for age, sex, and pubertal status.

Mean BMI‐SDS was significantly lower at diagnosis compared to premorbid status in the whole group (*F* = 81.1, *p* < 0.01). The decrease in mean BMI‐SDS was similar in prepubertal versus pubertal (*F* = 1.9, *p* = 0.16), male versus female (*F* = 0.47, *p* = 0.4), DKA versus non‐DKA (*F* = 0.72, *p* = 0.39), and non‐remitters versus remitters (*F* = 0.15, *p* = 0.6).

**FIGURE 1:**
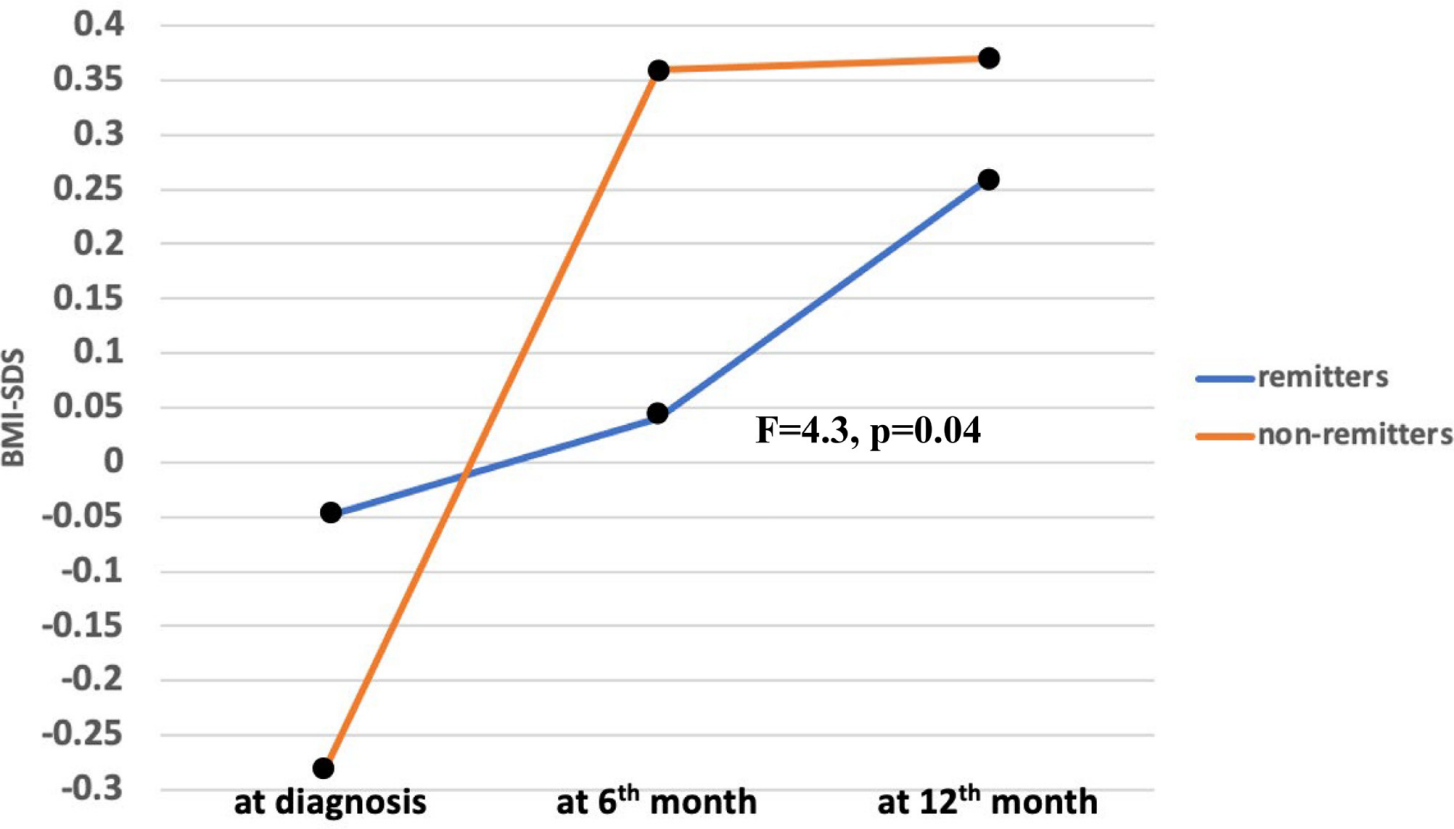
Early BMI‐SDS change in remitters versus non‐remitters.

**FIGURE 2:**
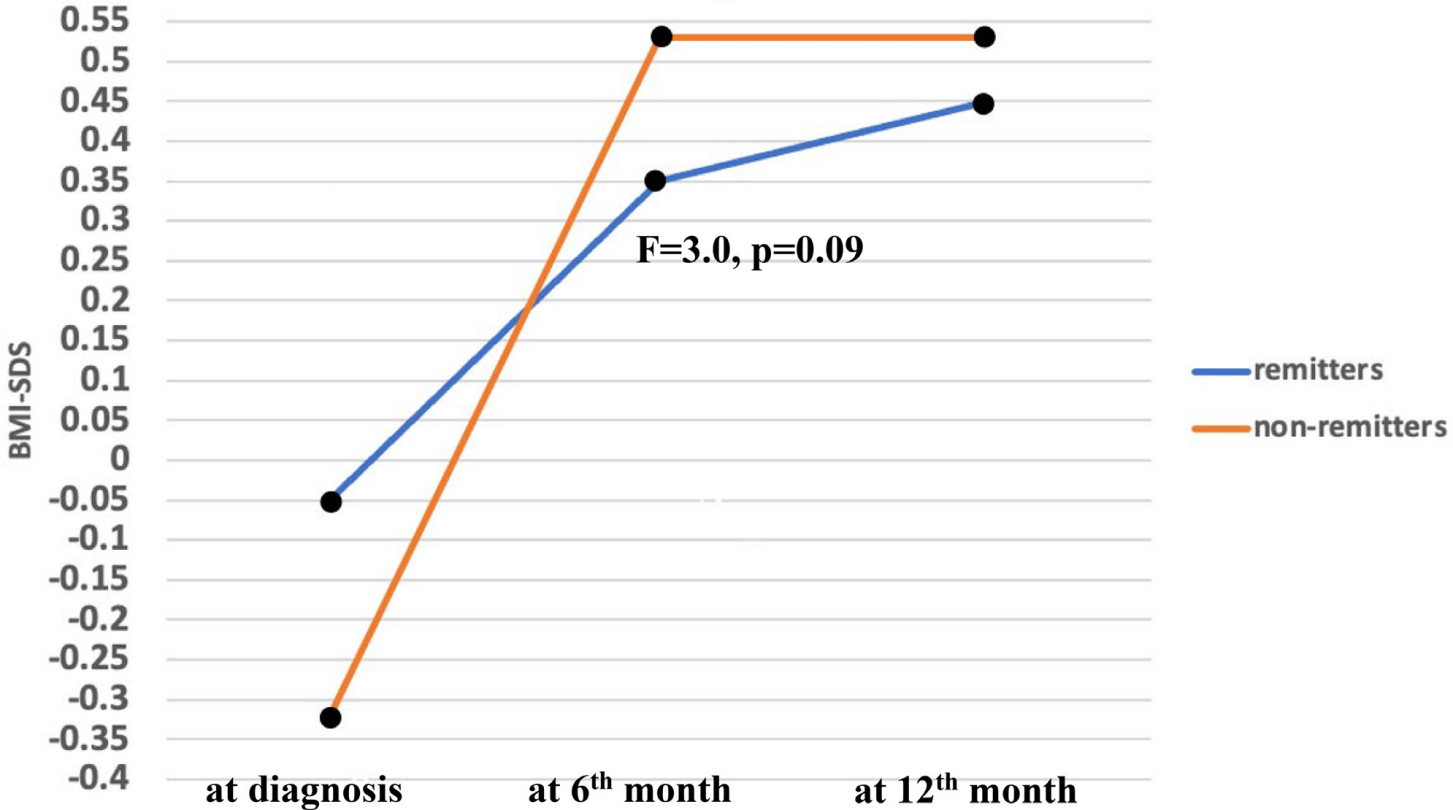
Early BMI‐SDS change in remitters versus non‐remitters after adjusting for puberty and sex.

After diagnosis, BMI‐SDS significantly increased in 6 months (*F* = 38.2, *p* < 0.01) in the whole group. The increase in mean BMI‐SDS did not differ according to pubertal status, sex, or presence of DKA at diagnosis (prepubertal vs. pubertal [*F* = 0.77, *p* = 0.38], male vs. female (*F* = 0.5, *p* = 0.48), DKA vs. non‐DKA [*F* = 0.08, *p* = 0.76]). There was no significant difference in terms of BMI‐SDS at each visit, ΔBMI‐SDS^1^ and ΔBMI‐SDS^2^ regarding pubertal status, sex, and presence of DKA at diagnosis (Table 3). However, mean increase in BMI‐SDS was higher in the non‐remitters in comparison to the remitters (*F* = 4.3, *p* = 0.04) (Table 1, Figure 1). This increase remained high in non‐remitters also after adjustment for pubertal status and sex (*p* = 0.09) (Figure 2).

**TABLE 3:** Comparison of BMI‐SDS at each visit, ΔBMI‐SDS^1^, and ΔBMI‐SDS^2^ regarding pubertal status, sex, and presence of DKA at diagnosis.

	BMI SDS			ΔBMI‐SDS^1^	ΔBMI‐SDS^2^
	At diagnosis	6 months	12 months		
Puberty					
Prepubertal	−0.17 ± 1.47	0.44 ± 1.07	0.51 ± 0.98	0.62 ± 1.06	0.006 ± 0.33
Pubertal	−0.19 ± 1.5	0.48 ± 0.96	0.46 ± 1.0	0.68 ± 1.01	0.06 ± 0.65
*p* value	0.94	0.87	0.83	0.78	0.58
Presentation					
DKA	−0.31 ± 1.48	0.34 ± 1.05	0.38 ± 0.90	0.65 ± 1.13	0.05 ± 0.61
Non‐DKA	0.007 ± 1.49	0.62 ± 1.01	0.65 ± 1.06	0.61 ± 0.92	0.03 ± 0.57
*p* value	0.29	0.18	0.17	0.85	0.83
Sex					
Male	0.11 ± 1.58	0.64 ± 1.04	0.65 ± 0.99	0.52 ± 1.08	0.02 ± 0.55
Female	−0.54 ± 1.29	0.23 ± 0.99	0.29 ± 0.93	0.77 ± 0.99	0.07 ± 0.64
*p* value	**0.02**	**0.04**	0.07	0.25	0.70

*Note*: Bold indicates significant value (*p* ≤ 0.05).

Abbreviations: BMI, body mass index; DKA, diabetic ketoacidosis; SDS, standard deviation score.

**FIGURE 3:**
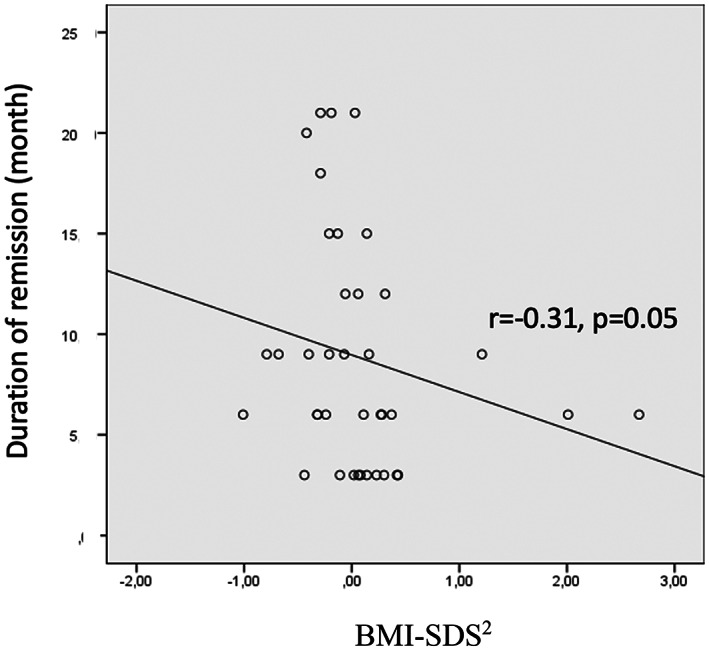
Correlation between BMI‐SDS^2^ and duration of PR.

The mean duration of PR was 8.9 ± 5.7 months which did not differ between male versus female, pubertal versus prepubertal patients, patients with DKA versus non‐DKA (9.2 ± 6.2 vs. 8.0 ± 4.1 months *p* = 0.44, 10.6 ± 7.5 vs. 8.6 ± 5.4 months *p* = 0.60, 8.6 ± 6.0 vs. 9.2 ± 5.3 months *p* = 0.71 respectively). Duration of PR was not correlated to clinical parameters [age, sex, puberty status, presentation status, BMI‐SDS (*p* = 0.61, 0.91, 0.90, 0.34, 0.46 respectively)] or laboratory parameters [blood glucose, pH, HCO3, insulin, C‐peptide, HbA1c (*p* = 0.32, 0.24, 0.29, 0.78, 0.26, 0.10 respectively)] at diagnosis. There was no significant correlation between duration of remission and BMI‐SDS at diagnosis/6th month/12th month and Δ BMI‐SDS^1^ (*p* = 0.1, 0.28, 0.06, 0.76 respectively). Only Δ BMI‐SDS^2^ was found to be correlated with the duration of PR (r = −0.31, *p* = 0.05) (Figure 3).

## 4 DISCUSSION

Patients with newly diagnosed T1DM can experience PR with the initiation of insulin therapy and decreasing glucose toxicity. The prevalence of PR in the current cohort was 47.5%, which is comparable to previous studies using IDAA1c criteria.^14^, ^15^, ^16^ However, various prevalence rates of PR were also reported.^17^, ^18^ This wide range may be due to the differences in country/race, treatment modality, and definition of PR.

In our cohort, intensive insulin therapy was followed by a significant increase in BMI‐SDS in the whole group, which was expected.^11^ The major finding in our study was that remitters and non‐remitters who have similar BMI‐SDS at diagnosis showed variation in weight gain during the early phase. Premorbid weight loss or hydration status may also affect the postdiagnosis change in weight. However, since the severity of disease (presence of DKA, HbA1c, pH, degree of dehydration), BMI‐SDS at diagnosis, and premorbid weight loss were not different between remitters and non‐remitters, it was suggested that remission may be associated with less weight gain in children with T1DM. Previous studies examining the relationship between BMI‐SDS at the time of diagnosis and PR rate suggested that higher BMI‐SDS at diagnosis may be associated with a higher rate of PR.^17^, ^19^ Higher resistance to insulin in individuals with a higher BMI‐SDS is supposed to accelerate overt hyperglycemia with an earlier clinical presentation before total beta‐cell loss. It is suggested that the remaining beta‐cell population in such individuals may facilitate the development of remission after insulin treatment. However, there are also other studies showing no difference in BMI‐SDS at diagnosis between remitters and non‐remitters.^15^, ^20^ Furthermore, it is well known that insulin requirement and insulin sensitivity are expected to be negatively correlated in patients with T1DM.^6^ Martin et al. reported change of BMI within the first year of diagnosis in a group of children with new‐onset T1DM. In their group, the initial BMI was higher in remitters; however, at 1 year follow‐up, the BMI was similar in both remitters and non‐remitters.^4^ This might be interpreted as an indirect evidence that remitters gain less weight than non‐remitters in the early phase of insulin treatment. However, there is no study analyzing the impact of early change in weight/BMI on development of remission. In the current study, we addressed this issue in a group of children with new‐onset T1DM. Remitters and non‐remitters, who had similar BMI‐SDS at diagnosis, showed variation in weight gain during the early phase which seemed to affect the development of PR, that is, less weight gain led to an increased likelihood for remission. Furthermore, duration of PR was found to be negatively associated with BMI‐SDS change between 6 and 12 months after diagnosis. These findings may be a reflection of the acceleration hypothesis in the PR phase, the core concept of which is that weight gain increases insulin resistance and consequently worsens glycemic control.^21^ In a recent longitudinal study, it was shown that patients with insulin resistance did not develop PR although their ß‐cell function reserve was better. This study provided evidence for the impact of insulin sensitivity rather than beta‐cell reserve on the pathophysiology of PR.^22^ The lower prevalence of PR in pubertal cases in our study can be accepted as another sign for the association of insulin sensitivity and PR.

The current study showed that prepuberty, male sex, younger age, and low HbA1c at the time of diagnosis were also positive predictors of PR. However, after adjustment, the only independent factor facilitating the development of PR was male sex. Previous studies investigating the epidemiology of remission in children with T1DM demonstrated that various parameters could affect the development of PR.^2^, ^23^, ^24^, ^25^, ^26^ In some publications, it has been reported that female patients with T1DM need more exogenous insulin than males during both the prepubertal and pubertal periods.^27^, ^28^ Furthermore, it is known that the insulin sensitivity of patients with T1DM differs in a wide range and is lower in female patients.^6^ The lower rate of remission in females in our study may also reflect the importance of insulin resistance in the pathophysiology of PR.

The major limitation of our study is related to its retrospective design; thus the effect of premorbid weight loss on postdiagnosis weight gain, insulin sensitivity indices, β‐cell reserve, and factors (eating habits, exercise, etc.) that may have an impact on weight gain could not be analyzed. However, this is the first study, as far as we have investigated the English literature, that shows the significant association between early weight gain after diagnosis and remission phase. Therefore, a prospective study with a large number of cases is expected to address this issue in more detail.

In conclusion, the results of our study suggest that rapid weight gain in the early stage of intensive insulin therapy may play a role in non‐remission, possibly due to insulin resistance caused by weight gain, and support the notion that insulin sensitivity plays an important role in the pathophysiology of remission. Interventions to prevent rapid weight gain and conserve insulin sensitivity in the early stages of T1DM may be beneficial for the development and maintenance of remission.

## FUNDING INFORMATION

This research received no specific grant from any funding agency in the public, commercial, or not‐for‐profit sectors.

## CONFLICT OF INTEREST STATEMENT

The authors declare no potential conflict of interest.
